# Assessment of factors influencing glymphatic activity and implications for clinical medicine

**DOI:** 10.3389/fneur.2023.1232304

**Published:** 2023-09-07

**Authors:** Adam Gędek, Dariusz Koziorowski, Stanisław Szlufik

**Affiliations:** ^1^Department of Neurology, Faculty of Health Sciences, Medical University of Warsaw, Warsaw, Poland; ^2^Praski Hospital, Warsaw, Poland

**Keywords:** glymphatic system, sleep, AQP4, Alzheimer’s disease, Parkinson’s disease

## Abstract

The glymphatic system is a highly specialized fluid transport system in the central nervous system. It enables the exchange of the intercellular fluid of the brain, regulation of the movement of this fluid, clearance of unnecessary metabolic products, and, potentially, brain immunity. In this review, based on the latest scientific reports, we present the mechanism of action and function of the glymphatic system and look at the role of factors influencing its activity. Sleep habits, eating patterns, coexisting stress or hypertension, and physical activity can significantly affect glymphatic activity. Modifying them can help to change lives for the better. In the next section of the review, we discuss the connection between the glymphatic system and neurological disorders. Its association with many disease entities suggests that it plays a major role in the physiology of the whole brain, linking many pathophysiological pathways of individual diseases.

## 1. Introduction

The human lymphatic system is an open system of vessels and ducts filled with lymph. Flowing through the peripheral tissues, the lymph collects unnecessary proteins, toxic substances and excess fluids and transports them through appropriate pathways, leading to their removal from the body (1). Lymphatic vessel density is related to the rate of tissue metabolism. The brain is characterized by a high metabolic rate (2), which entails the need for effective drainage and cleansing. Even so, it has no lymphatic vessels. They only occur between the meninges and are involved in improving the flow of the cerebrospinal fluid (CSF). However, they do not penetrate into the brain parenchyma (3–5). Therefore, one wonders how the correct volume of fluids in the central nervous system (CNS) is maintained and how the waste products and other substances are drained away. Until recently, this role was attributed to the passive movement of the cerebrospinal fluid. Recent reports have refuted this view and contributed to the discovery of a system that is functionally equivalent to the peripheral lymphatic system, which, due to the involvement of glial cells, was called the glymphatic system (6). The existence, though supported by various studies, remains in question due to multiple controversies. These are enhanced by the recent discovery of lymphatic vessels within the skull (3–5) and the fact that most of the research has been carried out on animals. Nevertheless, the concept of the glymphatic system seems consistent, and while findings have expanded our understanding of it, there is still a lot to be done. Several studies report on the relationship between the activity of the glymphatic system and neurodegenerative diseases (7) or the aging process (8). However, the system gained interest due to the discovery that it exhibits variable activity in relation to sleep and wake states (9). This allowed for a broader look at the physiological functions of sleep (10) and its hygiene, which (by influencing the cleansing of the brain) can potentially prevent the development of neurodegenerative diseases. Recent studies also indicate other factors contributing to the increase in glymphatic activity. Some of them may be subject to modification, e.g., by changing lifestyle. In this review, based on the latest scientific reports, we present the mechanism of action and function of the glymphatic system and show the role of sleep and other factors influencing its activity. In addition, we discuss the connection between the glymphatic system and neurological disorders.

## 2. Physiology of the glymphatic system

The glymphatic system is a highly specialized fluid transport system in the central nervous system. It enables the exchange of intercellular fluid of the brain, containing unnecessary metabolic products (and other substances, e.g., proteins responsible for the occurrence of dementia, such as amyloid beta or tau protein), with newly formed cerebrospinal fluid. As a result of its specific structure, it creates a route along which the liquid flows. Animal studies have shown that the cerebrospinal fluid present in the subarachnoid space flows into the grain tissue accompanying arteries in the surrounding perivascular space (the so-called Virchow-Robina - VRS) (6, 11–13). Initially it is located around the large, superficial meningeal vessels, which branch into penetrating arteries and penetrate deeper into the brain parenchyma, eventually dividing into capillaries (6). Similar fluid flow has been observed in humans, using MRI after intrathecal administration of a tracer (14, 15). The perivascular space is limited from the inside by the basal membrane of the vessels, and from the outside by protrusions of the glial cells–astrocytes. There are numerous water channels on them that allow the penetration of fluid from this space into the parenchyma of the brain, called aquaporin 4 (AQP4) (6). Such an arrangement of channels (polarization), enables real communication between the CSF and the parenchyma of the brain (16), and the mixing of fluids (CSF and ISF–interstitial fluid). While there have been reports rejecting the role of AQP4 (17), a meta-analysis of six independent experiments in recent years has confirmed that its misalignment or complete deletion inhibited the influx of markers and contrast agents in mice (18). A recent study confirmed these reports, indicating that deletion of AQP4 in mice resulted in increased accumulation of beta amyloid (Aβ) and tau protein in the brain (19). The polymorphism in the *AQP4* gene is also important (20, 21). SNP (single nucleotide polymorphism) influenced the accumulation of amyloid in the brain and the sleep quality of the study participants, which indicates reduced activity of the glymphatic system (21). In addition, it was found that in people suffering from idiopathic normotensive hydrocephalus and in the aging brain (i.e., in states where glymphatic activity is impaired), there was a loss of the perivascular location of aquaporin 4 (22–24). The movement of fluids within the brain parenchyma itself is subject to numerous discussions. It is probably possible due to the existence of two mechanisms: convective transport and diffusion (25). The simulations suggest that diffusion may be characteristic of small molecules and convective flow may be characteristic of larger molecules (26). This transport is influenced by such elements as pulsation of arteries (12) and arterial pressure (18). It is also positively correlated with decreased heart rate (27). Rodent studies document that fluid from the brain parenchyma enters the venous perivascular space around the vessels that drain blood from the brain. From here it can be removed in three ways. The first is the ethmoid plate, through which it finally enters the nasal mucosa, and then the cervical lymph nodes (28). The second way is through the spaces along the cranial nerves, such as the trigeminal nerve, the glossopharyngeal nerve, the vagus nerve, the spinal accessory nerve or the facial nerve (29). The third way is the recently discovered lymphatic vessels in the dura mater located around the dural venous sinuses (3, 5, 29, 30). It should be remembered that the mechanism of action of the glymphatic system is a relatively new discovery. The amount of experimental data is increasing, but many issues are controversial and subject to wide discussion (31).

## 3. The influence of sleep on the functioning of the glymphatic system

### 3.1. Sleep physiology

Sleep is a physiological, reversible state of the human body. Although it is a common and apparently obvious phenomenon, its neurobiology is complex (32). Over the years, the exact purpose of sleep has not been determined, although there are several valid theories (33). From 1953, thanks to the discovery by Aserinsky and Kleitman of the rapid eye movement (REM) phase, we know that it consists of two neurophysiological states that we are able to distinguish (34). In addition to the aforementioned REM phase, there is a non-rapid eye movement (NREM) phase. The gold standard used to monitor physiological sleep is polysomnography, which presented the exact architecture of sleep in certain phases using measurement of the brain’s electrical activity (35). The states of sleep and wakefulness are divided into wakefulness–stage W, NREM sleep - NREM (N1, N2, N3, and N4) and REM sleep. When falling asleep, a person falls through the successive stages of NREM until they fall into slow-wave sleep. During it, the metabolic rate, blood pressure, respiratory rate and temperature decrease. It is followed by REM sleep, which is characterized by the occurrence of dreams. The entire NREM-REM cycle lasts approx. 90 min. During one night we go through about 4–6 such cycles (assuming that the sleep lasts from 6 to 8 h). We know that the NREM phase is the moment when the body regenerates, but despite the passage of many years, the functional relationship of REM and NREM still leaves many questions unanswered (36). In addition, sleep patterns change over the years. Although this is often independent of factors such as comorbidities or medications, healthy elderly people are less likely to report sleep problems (37).

We are able to monitor the electrical activity of the brain during sleep using EEG (electroencephalogram). Although the analysis of electrical patterns can be difficult (38), basically, depending on the frequency, we can distinguish four types of electric waves corresponding to the respective states of sleep or wakefulness. Beta waves have a frequency of 14 to 30 Hz and reflect the involvement of the cerebral cortex in cognitive activity. Alpha waves with a frequency between 8 and 13 Hz are associated with a state of relaxation and reduced cognitive activity, but in the waking state. Theta waves have a frequency of 4 to 8 Hz and can be observed during light sleep and during hypnotic states. This corresponds to stages 1 and 2 of NREM. A different type of theta waves is involved in cognitive activity. Delta waves have a frequency of up to 4 Hz and are observed during sleep in the 3 and 4 stages of NREM (39, 40).

### 3.2. The influence of sleep on the activity of the glymphatic system

The first report on the influence of sleep on the functioning of the glymphatic system comes from 2013. Xie et al. used three groups of mice injected with fluorescent marker into the subarachnoid space (cisterna magna) to check its penetration in various states of somatic activity. These groups included awake mice, sleeping mice, and mice anesthetized with a mixture of ketamine and xylazine. Before the marker was administered, their condition was checked by assessing brain waves with EEG. Researchers observed an increase in intracellular volume by 60% in the group of natural sleeping and anesthetized mice compared to awake mice. The authors found that sleep accelerated the brain’s clearance of beta amyloid. The increase in flow through the glymphatic pathway and the increase in the volume of interstitial fluid occurred mainly during the more frequent occurrence of delta waves during sleep, corresponding to the NREM phase (9). Another research method was used by Achariyar et al. Using apolipoprotein E (apoE) as a marker, they conducted an experiment on animals in which they administered it to the choroid plexus. Then, using immunological diagnostic methods, its influx to the brain and clearance from the mouse brain were assessed. It was confirmed that the influx of CSF into the brains of sleep deprived mice is lower. When examining the distribution of apoE in the brain, it was found that depriving rodents of 3 h of sleep significantly reduced the distribution of apolipoprotein around arterial vessels (41).

In studies of glymphatic activity during sleep in humans, the measurement of the volume of the perivascular space was used. In one retrospective study, an association was found between sleep time and perivascular volume in patients with cerebrovascular disease (specific diagnoses were: transient ischemic attack (TIA; *n* = 7), acute stroke (*n* = 4), subacute/ chronic stroke (*n* = 4), and other cerebrovascular diagnoses (*n* = 6)). Sleep was assessed using polysomnography and Virchow-Robin space was assessed using high-quality 3D MRI. It was observed that total VRS, and basal ganglia VRS volume were negatively correlated with effective sleep (total sleep time divided by total time spent in bed). Furthermore VRS in the basal ganglia were negatively correlated with duration of sleep phase N3. This suggests that shorter sleep times are associated with ineffective brain drainage, which may lead to an enlargement of the perivascular space. The measure of WASO (time spent awake after the first episode of sleep, but before final awakening) was positively correlated with an increase in the volume of VRS, which suggest that sleep quality is of great importance in cleansing the brain (42). Similarly, in Yang et al. study, which included 398 patients after lacunar stroke, EPVS (enlarged perivascular spaces) in basal ganglia were associated independently with poor sleep quality, extended sleep latency, and reduced sleep duration. However, EPVS in white matter were also independently related to poor sleep quality, shorten sleep duration, and heightened sleep disturbances. The differences between the brain regions, possibly caused by different pathologies and anatomical structures, and possibly influenced by factors such as hypertension and age. EPVS in white matter may reflect cerebral amyloid angiopathy, whereas EPVS in basal ganglia may indicates hypertensive arteriopathy. In the future, longitudinal studies should be conducted to assess the relationship (43). Consistent results were obtained by Opel et al., who investigated the relationship between sleep, traumatic brain injury (TBI) and enlarged perivascular spaces. In this study, polysomnography and 3 T MRI were used. People with shorter total sleep times were found to have significantly greater total VRS volume. Such enlarged spaces have been found to be associated with developing amyloidopathy. In addition, it was observed that in patients who had suffered traumatic brain injury, the relationship between sleep time and EPVS was stronger (44).

Recent reports using the apparent diffusion coefficient (ADC) indicate that while in humans the mechanism and function of the glymphatic system may be more complex (compared to rodents), the main findings remain consistent. The authors, based on the water diffusivity hypothesis (assuming that the volume of CSF increases during sleep and, consequently, ISF) used diffusion magnetic resonance for the cortical assessment of ISF. Fast-ADC is more sensitive to changes in cerebral blood flow and slow-ADC reveals diffusion changes at the tissue level. In a sample of 30 patients, it was shown that sleep was associated with increased ADC in the dorsal cerebellum (slow-ADC) and left temporal pole (both slow, and fast-ADC), and decreased fast-ADC in right globus pallidus and insula, left thalamus, parahippocampus, and ventral cerebellum. Density of sleep awakenings was inversely related to ADC changes. When comparing 20 patients, there was no difference in ADC between wakefulness and overnight deprivation. This study suggests that there may be different diffusion patterns in various brain regions, depending on cell activity and neurotransmitters in specific areas. Furthermore, the correlation between ADC changes and awakenings/sleep quality suggests that they may more interfere with glymphatic function in humans, reflecting complex sleep patterns that were better controlled in mice (45).

Rainey-Smith et al. proved that AQP4 should be taken into account when analyzing the effect of sleep on the glymphatic system. Perhaps it is a factor that moderates the activity of the glymphatic system during sleep. Different variants of the aquaporin 4 gene correlated with sleep quality as measured by the Pittsburgh Sleep Quality Questionnaire (such as overall sleep quality, time to fall asleep, sleep duration) (21).

Norepinephrine is considered to be the key regulator of switching between sleep and wakefulness (46). It is the main stimulating neuromodulator, responsible for the increase in neuronal activity and the collection and processing of sensory information (47). The norepinephrine level is higher in the waking state and decreases in the sleep state. In adult mice brains, the administration of a mixture of adrenergic antagonists caused a decrease in the activity of the glymphatic system similar to the sleep state (9). In turn, rapid stimulation caused the release of norepinephrine and, consequently, a reduction in the intercellular space in the parenchyma of the brain. This led to an increase in resistance and a porter convective flow. In addition, norepinephrine reduced the production of CSF by the choroid plexus. It has recently been proposed that it can induce stasis in the glial pathway through microglia activation (48), which is negatively correlated with glymphatic activity in aged mice (49).

The above studies confirm that (as suggested by Xie et al.), the function of sleep may be the need to remove unnecessary metabolites in order to maintain homeostasis in the brain environment (9). Both the right amount of sleep and its good quality contribute to an increase in glymphatic activity, and thus cleansing the brain. Probably AQP4 plays a big role here. Even a small loss during sleep can affect its activity and contribute to the accumulation of unnecessary metabolic products or beta amyloid. The cleansing is most effective in phase 3 and 4 of NREM, when the brain is characterized by delta waves. Glymphatic activity is significantly correlated with the time of falling asleep or waking up.

### 3.3. The influence of body position during sleep on the activity of the glymphatic system

The discovery that the level of consciousness significantly influences the activity of the glymphatic system has led to further research hypotheses. In 2015, Lee et al. found that the position of the body during sleep plays an important role. In their experiment, they used three groups of rodents that were immobilized in appropriate positions: dorsal, abdominal and lateral. Using magnetic resonance imaging, they performed *in vivo* and *ex vivo* imaging, which showed that the activity of the glymphatic system is greatest in the lateral position and lowest in the abdominal. The authors note that primates (and, in fact, most mammals) sleep on their side, which may be due to an evolutionary process to ensure optimal functioning of the glymphatic system (50). In 1983, De Koninck et al. found the lateral position during sleep is chosen by people more often (51). These findings are confirmed by more recent, larger studies using more advanced measurement methods. Accelerometer records showed that the lateral position was the dominant sleep position for adults. Moreover, it was found that this dominance increases with age and BMI (52).

In 2017 Lundgaard et al. used a different research approach, focused on studying the level of lactate in the brain in three groups of mice (53). This acid is synthesized in astrocytes from glucose and glycogen, ensuring an adequate level of energy for neurons. This synthesis is stimulated by norepinephrine (54). In one group, the mice were arranged non-physiologically–upside down. In the second one, mice lacking AQP4 (AQP4 knockdown) were used, and in the third, the activity of the glymphatic system was inhibited with acetazolamide. In all groups it was observed that the level of lactic acid during sleep remained constant, while physiologically it should decrease. Depriving the mice of sleep also resulted in lactate accumulation in the brain, and no increase was seen in the cervical lymph nodes. The experiment showed that lactate is drained from the brain during sleep and can be considered a marker of glymphatic activity. It also suggested that body position is important to glymphatic activity (53).

It was decided to test the relationship between sleep position and the glymphatic system in humans. It turned out that patients suffering from neurodegenerative diseases more often spend the night in the dorsal position (sleep on their backs) than people with normal cognitive functions. There was no difference in the amount of position changes during sleep between the groups (55). There was an attempt to explain this mechanism by Simka et al. by conducting a pilot study on blood flow through the jugular veins. By ultrasound examinations of 3 healthy people and 2 patients with abnormal venous valves, he found differences in the collapse of the vessel walls in the sitting position and in the lying position on both sides. In healthy people lying on their side, one jugular vein remained open and the other partially collapsed. This was not observed in people with valve defects whose internal jugular veins were open. In the dorsal position, the lumen of both veins was reduced. Researchers suggest that the lateral position is associated with the optimal venous circulation and thus the proper functioning of the glymphatic system (56). Simka et al. also suggest that one should look at chronic cerebrospinal venous insufficiency, which is a new disease entity observed in multiple sclerosis, Parkinson’s disease and Alzheimer’s disease (56).

### 3.4. Sleep deprivation

The effect of sleep deprivation on the glymphatic system was also investigated. Using continuous theta stimulation (cTBS), glymphatic activity was compared in normal mice and animal models of sleep deprivation. Using two-photon *in vivo* imaging, it was found that sleep deprivation reduced fluid inflow to the perivascular space, and immunofluorescence showed that it distributed AQP4 polarization. In addition, the open field test revealed that it triggered anxiety behaviors. cTBS led to a recovery in these deviations, which also indicates that it can be used to protect the dysfunction caused by sleep disorders (57). Another study showed that sleep deprivation increases the accumulation of beta-amyloid in the human brain. Shokri-Kojori et al., using PET and ^18^F-florbetaben, assessed the beta amyloid load in healthy people after a full night of sleep and a sleepless night. A sleepless night has been shown to increase beta amyloid levels in the hippocampus and thalamus (58). Both sleep deprivation and partial sleep loss negatively affect cognition. The relationship between the dysfunction between sleep–wake states and beta amyloid deposition seems to explain the glymphatic system and the function of astrocytes. This is especially important because sleep disturbances are an early predictor of neurodegenerative diseases (59).

## 4. Circadian rhythm and the influence of light

Sleep, which affects glymphatic activity, is under the control of circadian rhythms. Recent reports indicate that the activity of the glymphatic system itself may also be subject to the circadian rhythm, and thus not only depend on the state of stimulation. In a study by Hablitz et al., the peak glymphatic activity in rodents was at noon, when the mice were most asleep. However, the existence of endogenous rhythms of glymphatic activity was found throughout the day. CNS drainage from the cisterna magna to the cervical lymph nodes varied with the time of day and was the opposite of the influx of lymph fluid to the brain. These rhythms also persisted under changing light. This suggests that all CSF distribution depends on the time of day. Increased influx of CSF into the brain was associated with a greater polarization of AQP4, which probably supports this rhythm. AQP4 knockdown mice showed no day/night variation in glymphatic influx (60). Previously, Taoka et al. conducted two experiments. First, using dynamic MRI, they investigated whether a gadolinium-based contrast agent injected intravenously into mice penetrates into the cerebrospinal fluid. Secondly, using inductively coupled plasma mass spectrometry, it was examined whether the concentration of the contrast depends on the time of administration during the day and whether the anesthesia affects its distribution in the brain. Four groups of mice were injected with gadolinium, divided according to the time of injection (morning, late afternoon) and anesthesia (absent, short, long). It was administered 8 times for 2 weeks. Five weeks after the last injection, the tissue contrasts levels corresponding to the glymphatic activity were assessed. It turned out that glymphatic clearance was most effective in mice receiving contrast in the morning, and anesthesia increased this effect. Since mice are nocturnal animals, this seems to confirm the findings so far. The authors suggested that the circadian rhythm is important for glymphatic activity (61). Another study used dynamic magnetic resonance imaging to evaluate redistribution of the contrast agent throughout the brain in conscious rats. The inflow and parenchymal distribution were assessed in relation to the inflow of light. The highest inflow and distribution was demonstrated in the dark phase, and the lowest in the light phase. The authors also showed the inflow to precise regions of the brain and the relationship of fluid redistribution with temperature and vascular density (62). We know that biological rhythms such as light–dark or sleep–wake and other physiological rhythms are closely related. However, more research is needed to understand the exact mechanism that connects these rhythms and how they relate to glymphatic activity.

## 5. Anesthesia and glymphatic system

Recently, Hablitz et al. found that delta waves occurring during anesthesia are particularly correlated with high CSF influx into the brain (27). While beta waves showed a little predictive ability, neither gamma nor alpha waves correlated with the influx of a special market into the brain. Researchers also found relationships between glymphatic activity and the circulatory system. Heart rate under anesthesia showed a strong inverse correlation with lymphatic influx. Neither respiratory rate nor systolic blood pressure correlated with CSF market influx. This shows an interesting to understand axis of the dream-heart-glymphatic system. The key issue in examining the glymphatic system under anesthesia may be the selection of an appropriate esthetic. Although the ketamine/xylazine system appears to be the best in terms of glymphatic activity, the most common uses in clinical practice are isoflurane (iso) and dexmedetomidine (dex). While iso adversely affects glymphatic flow, dex improves it (27). This is in line with the studies carried out by Beneviste et al. They showed that in rats anaesthetized with dexmedetomidine with a low dose of isoflurane, lymphatic flow increased by 32% compared to mice anaesthetized with isoflurane alone. Comparing the electrical activities of the brain with EEG in both cases, it was observed that delta waves and sleep spindles were more common in the dex/iso group. It was also noted that in the hippocampus, clearance was six times more effective (63). It has previously been found that isoflurane anesthesia in mice increases beta amyloid deposition *in vivo* and is likely to be involved in promoting the development of Alzheimer’s disease (64). An *in vitro* study confirmed that isoflurane increases the oligomerization and cytotoxicity of AD-associated peptides. Apart from the general mechanism of the neurotoxicity of this substance, this suggests a connection with neurodegenerative diseases (65). Despite the disclosure of the harmfulness of inhalation anesthetics, some issues remain unclear and more research is needed to elucidate neuro-toxicity and the relationship with neurodegenerative diseases (66). Recently, Xue et al. conducted an experiment in mice using a novel MRI T1 mapping approach as an alternative to quantifying glymphatic transport. Increased glymphatic flow in mice anaesthetized with ketamine/xylazine relative to isoflurane was confirmed (67). Lilius et al. also confirmed that dexmedetomidine increases the activity of the slow wave in the EEG. This effect increased the exposure of the rat brain to oxycodone, naloxone, and IgG-size antibodies that were administered intrathecally. This discovery, therefore, may be an important step toward the effective administration of intrathecal drugs (68). The report on dex and iso was recently confirmed by Ozturk et al. (69). Cognitive impairment is associated with long-term sedation in intensive care units and general anesthesia during surgery, and patients receiving inhalation anesthetics are at risk of delirium. Dexmedetomidine has been shown to reduce the incidence of delirium (compared to placebo). Although the role of the glymphatic system in cognitive disorders and delirium has not yet been described, the authors suggest that it may be influenced by the mechanism of cleansing the brain from unnecessary substances that is stimulated by dex (27).

Gakuba obtained opposite results. While administering anesthetics, he found a weakening of the glymphatic system’s function in relation to the waking state (70). The reason was probably the use of other anesthetics (isoflurane and ketamine without the addition of xylazine, a beta adrenergic agent). In light of the earlier cited studies, this seems to be a significant change. However, it cannot be ruled out that the states of anesthesia are characterized by a different glymphatic activity than sleep (71).

## 6. Other factors influencing the glymphatic system

### 6.1. Omega-3

Current scientific evidence supports a link between Alzheimer’s disease and omega-3 polyunsaturated fatty acids. Observational epidemiological studies show that these acids can positively influence cognition in early Alzheimer’s disease (72). They probably also protect against its development (73). So far, only two studies have been conducted to analyze the effect of omega-3 polyunsaturated fatty acids on the functioning of the lymphatic system in the brain. In 2016, Ren et al. conducted an experiment administering fish oil (PUFAs) to transgenic mice. It has been found that this action significantly increases the interstitial clearance of beta amyloid and prevents the formation of its aggregates in the brain. This phenomenon was not observed in mice lacking AQP4 (AQP4 knockout), suggesting a relationship of this protein to promoting the activity of PUFAs. Polyunsaturated fatty acids have been found to inhibit astrocyte activation and to preserve the polarization of AQP4 in the affected region of the brain (after injection of amyloid beta). The authors suggest that n3-PUFA supplementation may delay or prevent the development of Alzheimer’s disease by improving glymphatic transport and reducing beta amyloid aggregation (74). This discovery and the proven relationship between the glymphatic system and traumatic brain injury (TBI) were used by Zhang et al. Using murine TBI models, they were able to establish that the administration of fish oil alleviated the development of neurological disorders. Moreover, it prevented the accumulation of beta amyloid and facilitated the removal of tracers after TBI. Omega-3 restored the expression and polarity of AQP4 that had been disrupted by brain damage. It also had a protective function on the blood–brain barrier. The authors explain that omega-3 supplementation has a neuroprotective effect with TBI by maintaining waste disposal and substance exchange in the brain and beyond at an appropriate level (19). Thus, the use of polyunsaturated fatty acids could become a cheap and effective prospect for improving glymphatic flow in the brain thanks to its influence on aquaporin 4. Even though omega-3 fatty acids appear to improve glymphatic activity, clinical trials and preclinical studies have shown that they have neuroprotective effects primarily by minimizing neuroinflammation (75, 76). There is a need for future research regarding the relationship between neuroinflammation and the glymphatic activity, and the effects of omega-3 on these processes (77).

### 6.2. Physical activity

An active lifestyle and increased physical activity are associated with a lower risk of developing Alzheimer’s disease (78). Experiments on animals indicate that this is another factor that stimulates the work of the glymphatic system. A study in mice showed that voluntary exercise (such as spinning the wheel) increased glymphatic clearance as well as the expression and polarization of AQP4 in the brains of animals. As a result, amyloid plaques accumulate less in the CNS. These mice also increased the number of dendrites, dendritic spines, and synaptic densities that are involved in synaptic formation and thus directly affect neuronal plasticity. There was also less microglia activation (49). The increase in glymphatic activity during voluntary exercise by mice was comparable to that observed with ketamine/xylazine anesthesia (79). These reports suggested treatments that target aquaporin 4 activity (such as aerobic exercise) as a new way to combat Alzheimer’s disease (80).

### 6.3. Alcohol

Lundgaard et al. were the first to investigate the short-term and long-term effects of alcohol on the glymphatic system. They administered low, medium and high doses of alcohol to mice and then took a brain fragment (30 min or 24 h from the last dose after injection into cisterna magna depending on the group) and assessed by microscopic examination. It was observed that a medium and a high dose of alcohol inhibited glymphatic activity, while a low dose improved its activity. The decrease in blood flow may be related to a decrease in cardiac output. Chronic alcohol administration for 30 days //at a medium dose decreased lymphatic activity, while at a low dose–it improved it insignificantly. This may explain the higher rate of dementia among drinkers (81). Also, Cheng et al. observed that a low dose promotes metabolite drainage through the glymphatic pathway while administering ethanol to rats. This was related to the stimulation of the endothelium of the cerebral arteries and smooth muscle cells by ethanol. The reactive expansion and contraction of vascular endothelial cells and smooth muscle regulated the diffusion and clearance of metabolites (82). Liu et al. reached similar conclusions after administering high and medium doses of alcohol. They found that it may be related to beta-endorphin release and reduced vascular pulsation. In turn, chronic moderate alcohol administration led to astrocyte activation and loss of AQP4 polarity. These mechanisms may contribute to cognitive dysfunction and dementia in alcoholism. The role of alcohol is extremely important because it impairs the glymphatic system irreversibly (83).

### 6.4. Stress

Although short-term stress plays an adaptive role in the human body, chronic stress is a factor in the development of Alzheimer’s disease. It causes the deposition of beta amyloid in the brain. Mice exposed to stress showed less glymphatic flow (influx and efflux), loss of aquaporin 4 polarity and fewer astrocytes. It is related to the interaction of glucocorticosteroids on their respective receptors. Mifepristone (a glucocorticoid receptor agonist) was found to improve glymphatic clearance by restoring the polarity of AQP4 (84).

### 6.5. Gender

Recently, Giannetto et al. noticed that there were no differences in glymphatic influx in different brain regions of mice by gender. This is puzzling since neurodegenerative disorders are more common in women. Perhaps this is due to additional sex-specific comorbidities (chronic stress, protein misfolding, traumatic brain injury, and others) that may increase the risk of developing proteinopathy (85).

### 6.6. Hypertension

Mortensen et al. showed that glymphatic transport is disturbed in both early and advanced states of hypertension. Using a rat model of hypertension, they measured CSF flow and followed the influx and outflow of lymphoid tissue with contrast-enhanced dynamic magnetic resonance. This observation demonstrates the value of vessel care in terms of its effect on the brain, and could provide a starting point to elucidate the relationship between vascular disease and Alzheimer’s disease (86). Recently, Kikuta et al. showed, that the perivascular spaces were significantly lower in the hypertension patients group, compared to healthy controls. These results emphasize the role of hypertension in glymphatic dysfunction (87).

### 6.7. Other factors

Zhang et al. demonstrated that intermittent fasting (IF) reduces the ratio of AQP4-M1/M23, which restores AQP4 polarity in an animal model of Alzheimer’s disease, causing a decrease in brain amyloid-beta accumulation. These suggest a positive influence of IF on glymphatic activity. Furthermore, β-hydroxybutyrate may mediate in this process, in which miR-130a and histone deacetylase 3 may also be involved (88). Injection of lipopolysaccharide (LPS) into the cisterna magna of mice decreased perivascular flow and marker penetration into the brain, although no differences were detected, for example, in AQP4 polarization. It was found that acute exposure to bacterial endotoxin causes a significant reduction in CSF distribution (89). In addition, high serum uric acid levels have been associated with enlargement of the perivascular spaces in patients with lacunar stroke (90).

**Figure 1:**
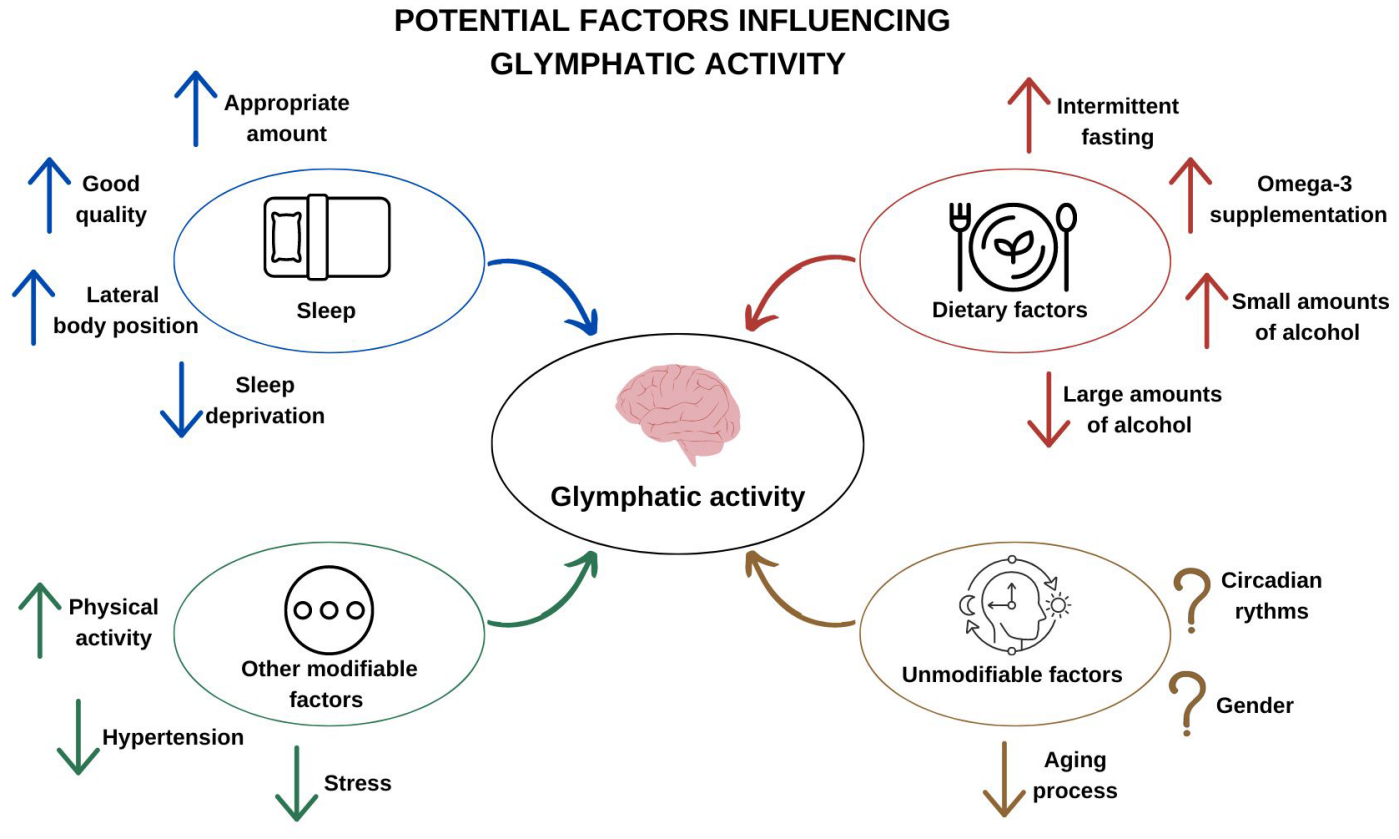
Potential factors influencing glymphatic activity.

A summary of potential factors influencing glymphatic activity is presented in Figure 1.

## 7. Glymphatic system–relationship with neurological diseases

Although the glymphatic system hypothesis has been tested primarily in animals, the first studies involving humans have recently been carried out. Impaired glymphatic clearance and abnormalities in interstitial fluid dynamics appear to be an important common aspect in the pathogenesis of several diseases that we can classify as Central Nervous System interstitial fluidopathies (91). In the following subsection, we present the current state of knowledge connecting the glymphatic system with neurological disease entities taking into account, first of all, the latest clinical reports.

### 7.1. Alzheimer’s disease (AD) and neurodegeneration

Due to its involvement in cleansing the brain, the activity of the glymphatic system was measured in relation to neurodegenerative diseases. Preclinical studies have shown that it is involved in clearing beta amyloid from the brain (6, 92). Its insufficiency preceded the formation of significant Aβ deposits. It has therefore been suggested that it may be an early biomarker of Alzheimer’s disease (92). The deletion of aquaporin 4, which is a key element of the glymphatic system, also promoted the accumulation of Aβ and contributed to an increase in cognitive impairment (6, 19, 93). It was also influenced by polymorphism in AQP4 (21). This channel has also recently been shown to be involved in the removal of tau protein from the brain. It was observed that animal models of tauopathy were characterized by an abnormal polarity of aquaporin 4, accompanied by an impaired exchange between the cerebrospinal fluid and interstitial fluid (94). It has also been shown that apolipoprotein E (ApoE), which is essential for the proper functioning of neurons, is delivered to the brain with the use of AQP4 (41). ApoE strongly influences the aggregation of amyloid beta in the brain and its clearance (95). Decreased CSF influx into the brain parenchyma and impaired clearance were also observed in an animal model of vascular dementia (96). Moreover, Kress et al. proved that the glymphatic system is more active in young animals. Age-related disturbance in aquaporin 4 expression correlated with lower tracer uptake by the brain parenchyma. In addition, the beta amyloid removal process was more efficient in young animals (16). Similarly, among humans the incidence of cognitive impairment and dementia increases with age. While the above reports come mainly from animal experiments, recent human studies seem to support these findings. MRI showed that in patients with dementia and abnormal intracranial pressure, contrast drained from the brain more slowly. Abnormal CSF drainage was probably associated with dysfunction of the glymphatic system (15). In addition, genetic variation in AQP4 in humans has been found to affect the relationship between beta amyloid accumulation and sleep (21).

Incorporating the current knowledge into the concept of the glymphatic system relatively recently means that finding appropriate research methods to assess its activity in humans remains a challenge. So far, various imaging techniques have been proposed, the development of which is an opportunity for a better understanding of the brain glymphatics (97–99). Choosing the best method is difficult because both in clinical work and research, the use of invasive methods that interfere with the CNS environment raises doubts, and the contrast-based imagining methods have practical limitations. Therefore, an interesting perspective is magnetic resonance imaging using the DTI-ALPS method (diffusion tensor image analysis along the perivascular space). The ALPS index represents the perivascular space and takes into account the diffusivity in the area of projection and associative fibers. Higher ALPS means greater water diffusivity along the PVS (perivascular spaces). This indicator seems to reflect glymphatic activity, although due to its novelty it requires further research (100). While it is a widely used method in studies of the glymphatic system, its accuracy should be confirmed and validated in future studies. It was first proposed for use in the context of the glymphatic system in 31 AD patients. A positive correlation was observed between ALPS and the results obtained by the subjects in the MMSE (Mini-Mental State Examination) scale (101). Similar results were observed in patients in a mixed cohort (AD, MCI, people without cognitive impairment) who underwent MRI. DTI-ALPS showed a positive correlation with the results achieved by patients in the MMSE and ADASCog11 (Alzheimer Disease Assessment Scale–Cognitive Subscale) scales (102). DTI-ALPS also correlated with the results obtained in the ECog (Everyday Cognition), CERAD-NB (Consortium to Establish a Registry for Alzheimer’s Disease Neuropsychological Battery) scales and gray matter volume in people over 60 without dementia (103). In addition, Zhang et al. reported that lower ALPS is associated with hypertension, venous disorders, and female gender (104). Recent studies confirmed that patients with AD had significantly lower ALPS index than healthy control (105–107). In addition ALPS index correlates with biomarkers of AD and cognitive dysfunction (106, 108, 109). Another method of imaging brain glymphatics used in human research, also based on magnetic resonance imaging, is the assessment of the perivascular spaces (PVS), which can be observed using this method when they have widened (EPVS) in specific regions of the CNS. The severity of EPVS in basal ganglia has been shown to correlate with age in an older healthy population, and it has been suggested that widening at the semiovale center may affect beta amyloid deposition, especially in cerebral amyloid angiopathy (110, 111). A recent study in a healthy population found that EPVS in the semiovale center was associated with tau accumulation, but not with beta amyloid deposition (112). Similarly, Banerjee et al. reported that although perivascular spaces are associated with AD, they do not affect amyloid burden (113). In a recent study, no correlation was found between EPVS and cognitive functions (114). It has been proven, however, that the perivascular spaces are influenced by various factors, such as gender and body weight, which suggests a relatively high inter-individual variability (115). The relationship between EPVS and Alzheimer’s disease is unclear and requires further research. Perhaps this is not an appropriate indicator to measure glymphatic activity, or the existing gaps in the current knowledge do not allow us to explain the discrepancy in these results.

### 7.2. Parkinson’s disease

While the interest in the glymphatic system initially focused on Alzheimer’s disease, research has also been conducted to reveal its association with Parkinson’s disease (PD). There are numerous similarities in the pathogenesis and the image of both of these neurological disease entities in which pathological proteins accumulate. A hallmark of PD is dopaminergic dysfunction, which can cause sleep deprivation, which is a key player in brain glymphatics. This relationship may influence alpha-synuclein deposition and disease development (116). Initially, the relationship between the glymphatic system and PD was confirmed using preclinical models. It was observed that AQP4 deficiency in mice increased their susceptibility to 1-methyl-4-phenyl-1,2,3,6-tetrahydropyridine (MPTP), used as a toxic model of PD in animals. It was associated with increased dopaminergic degeneration, contributed to an increase in the number of reactive astrocytes and influenced inflammatory processes (117). Using a murine model of mutant alpha-synuclein overexpression, it was shown that decreased marker influx was associated with increased aggregation of alpha-synuclein in the perivascular space and impaired AQP4 polarization in the substantia nigra, suggesting a dysfunction of the glymphatic system (118). Recently, a large study has been conducted showing that meningeal drainage dysfunction may contribute to the development of PD. Using dynamic contrast-enhanced MRI (DCE-MRI), decreased flow through the meningeal lymphatic vessels and delayed deep lymph nodes flow in patients with idiopathic disease (iPD) were observed, comparing these results with patients diagnosed with atypical parkinsonism. With intravenous injection of gadobutrol, carotid perfusion and lymphatic drainage in superficial lateral cervical lymph nodes were found to be the same in both cohorts. These results suggest that glymphatic clearance was reduced in patients with iPD, which was not found in the group of patients with atypical parkinsonism. Perhaps DCE-MRI may be an imaging marker that differentiates iPD and atypical parkinsonism, as distinguishing between these disease entities is not always easy. Moreover, in the same study it was observed that mice injected with alpha-synuclein also have delayed meningeal drainage, and blocking the flow through the meningeal lymphatic vessels exacerbates alpha-synuclein pathology and increases motor and memory symptoms of the disease (119). Analysis of MRI images of 470 people associated with parkinsonism (including 179 people with idiopathic PD form, 67 with family form, 104 from carriers and 84 from healthy people) showed a significant increase in the volume of the perivascular space on a global and regional scale, suggesting that sick and healthy people differ in the volume of these spaces in the medial orbitofrontal region and the banks of the superior temporal regions (120). Using perivascular diffusion tensor imaging (DTI-ALPS), it was shown that ALPS was reduced in patients with PD versus patients with movement disorders without proteinopathy, who were selected as controls because of the similar need for sedation during the study. This corresponds to a reduced perivascular fluid flow. In this study, no association was observed between ALPS and MCI (121). However, an earlier study involving 271 patients showed that enlargement of basal ganglia PVS is associated with cognitive decline in PD patients and may be a useful clinical marker (122). In another study, using resting state fMRI and global blood-oxygen-level-dependent (gBOLD), which can be used to assess glymphatic function, significantly lower values of this index were observed in patients with Parkinson’s disease who showed cognitive impairment, compared to the control group or patients with PD, but without cognitive impairment. However, no relationship was observed with the results of the UPDRS scale (123). Moreover, Chen et al. observed that ALPS is significantly lower in PD patients than in healthy controls and suggested that there may be a relationship between glymphatic dysfunction and increased oxidative stress (lower ALPS index correlated with an increase in nuclear DNA in plasma) (124). Recently, it has been observed that the DTI-ALPS ratio is lower in patients with late Parkinson’s disease, however, no clear differences were noted between early stage PD and the control sample. However, it was possible to establish that ALPS correlated positively with cognitive functions in the early stage and vice versa – with age in the advanced stage (125). Lower ALPS rates in Parkinson’s patients compared to healthy controls have been confirmed in later studies (126–128). More research is needed on glymphatic flow at specific stages of PD. It is worth noting that there is also a documented relationship between AQP4 and expression and alpha-synuclein accumulation and that its expression, as well as AQP1, can modify alpha-synuclein deposition in the cortex of new patients, as demonstrated in a post-mortem study (129). AQP4 may be an important compound that connects and influences various molecular pathways, such as activation of astrocytes, microglia, changes in alpha-synuclein function and inflammatory factors, more on this in the review by Tamtaji et al. (130). Perhaps the glymphatic system is a key player in the inflammatory processes underlying the development of Parkinson’s disease.

### 7.3. Idiopathic normal pressure hydrocephalus

Idiopathic normal pressure hydrocephalus (iNPH) is the most common hydrocephalus in adults, affecting 10% of dementia patients. Clinically, it includes the characteristic triad of symptoms: ataxia, dementia, and urinary incontinence. Although lumbar puncture, performed for therapeutic purposes, improves gait, it often does not affect cognition. The pathogenesis of this disease is unknown, and diagnosis is made on the basis of clinical and imaging features (131). The first clinical reports on the relationship of iNPH with the glymphatic system come from 2017, when Ringstad et al. decided to assess glymphatic activity using MRI, after prior intrathecal administration of gadobutrol to sick and reference patients. It was observed that in iNPH patients the clearance of gadobutrol was delayed. In addition, it was driven by the pulsating movement of the intracranial arteries. It was also observed that glymphatic enhancement peaked at night in both tested groups. This study also showed a reduced flow in the entorhinal cortex (ERC), which formed the basis for another clinical trial (132). Two years later, similar results were obtained, confirming the hypothesis that impaired glymphatic function in the ERC and subcortical white matter in the ERC impedes substance clearance and contributed to a decline in cognitive functions. It is probably the interface between the neocortex and the hippocampus (133). Glymphatic MRI has been proposed to detect dementia at an early stage, but more research is needed to support this hypothesis (22). Absorption through the choroid plexus and slower clearance in iNPH patients has been observed after the injection of gadobutrol into the CSF. Since this structure has been shown to be involved in beta-amyloid clearance, it may indicate neurodegeneration (134). Delayed fluid clearance has also been observed in the visual pathway (135). Another research approach combining the glymphatic system with iNPH involved the use of an MRI diffusion tensor using DTI-ALPS (136). A reduction in the ALPS index was observed in patients with NPH. Moreover, it was lowered in patients who did not respond well to treatment and correlated with the corpus callosum angle reflecting the severity of the disease (137). It was recently reported that the mean number of vascular spaces seen was greater in the iNPH group than in the semiovale center and the basal ganglia groups, relative to the control group (138). Other evidence linking iNPH to the glymphatic system is the results of studies on aquaporin. A reduction in AQP4 in astrocytes (and Dp71) in the brain parenchyma of iNPH patients has been demonstrated (97). These results were confirmed in another study, concluding that restoring the appropriate AQP4 polarity may be a viable therapeutic option. In addition, a correlation was observed between the reduction in the correct polarity of AQP4 and the percentage of pathological mitochondria in patients with iNPH (139). A significantly increased incidence of pathological mitochondria in endfoot of astrocytes has also been observed in idiopathic intracranial hypertension (140). The role of the glymphatic system in the pathology of iNPH is also supported by the association of this disease with AD. They are often coexisting units (141), and patients with hydrocephalus more often develop AD (142), which may suggest a common pathogenesis of these disorders. Luikku et al. even propose performing a cortical biopsy during valve insertion to diagnose AD in patients with iNPH (142). In addition, sleep problems are common in iNPH, a feature common to both AD and glymphatic system dysfunction (143). Recently, Kikuta et al. showed, that ALPS index was significantly higher in the group after lumboperitoneal shunt surgery than in the pre-operative group, and suggested that glymphatic function recovery occurs following this surgery (144).

### 7.4. Traumatic brain injury

A link between traumatic brain injury and the glymphatic system was suggested shortly after its discovery. This seems logical from a glymphatic perspective, since patients after TBI often report sleep problems, and many of them develop neurodegeneration (145). Moreover, there is a relationship between amyloid beta and TBI, which may, on the one hand, play a role in the pathophysiology of trauma and, on the other hand, be a predictor and biomarker at different stages of trauma (146). Already Plog et al. suggested that the glymphatic system can help find TBI biomarkers by taking part in their transport into the blood (147–149). In 2014, Iliff et al. reported that after TBI, glymphatic system function in mice decreased by 60% and was maintained for about a month after severe and moderate trauma. In AQP4 knockout mice, the decrease in activity was more pronounced, which promoted neurodegeneration (150). Although the level of overall AQP4 increased after the injury, its localization changed–a decrease in its expression on the endfoot of astrocytes was observed. This applied to both mild and moderate injuries. Additionally, reactive astrogliosis was observed after moderate trauma (151). This polarization was less impaired in mice that has the adenosine A2A receptor deactivated (152). Restoration of AQP4 polarity may be an effective, innovative therapeutic method that has been validated in rats (153). However, it is not easy, especially since the results of research on AQP4 are unclear. Although it is known that it plays an important role, e.g., in the pathophysiology of brain edema after TBI, the effects of inhibition or knockout in animal models can be contradictory, depending largely on the model used, the characteristics of the injury or the study design (154). Recently, Park et al. confirmed that patients with TBI had significantly lower ALPS index compared to healthy controls (155). However, larger-scale and longer follow-up studies are warranted.

### 7.5. Vascular diseases–ischemic stroke, subarachnoid hemorrhage

Research on the relationship between the glymphatic system and ischemic stroke is inconclusive. Cerebral edema is an unfavorable clinical phenomenon that we can observe after a stroke. Initially, using a mouse model, it was shown that ligation of the internal carotid artery leads to a reduction in perivascular inflow, which confirmed the role of arterial pulsation in glymphatic flow (12). A year later, using MRI in mice injected with the marker, it was observed that ischemic stroke, as well as subarachnoid hemorrhage, impaired glymphatic perfusion, and administration of plasminogen activator improved glymphatic flow. However, it did not change with carotid ligation. The authors suggest that this result, contrary to the previous study, could be related to impaired pulsation of the arteries or obstruction of the perivascular space (156). Magnetic resonance imagining after intrathecal contrast administration in mice has shown that after an ischemic stroke, damage to the glymphatic system in the areas of secondary degeneration–the substantia nigra and the ventral nucleus of the thalamus (157) takes place. In another study, expansion of the perivascular spaces and an increase in glymphatic flow were observed in an animal model of ischemic stroke. It has been suggested that ischemia can lead to depolarization that drives the glymphatic flow that contributes significantly to acute edema (31). Thus, it is unclear whether an ischemic stroke disrupts the glymphatic flow or, on the contrary, it is involved in the inflow of fluid and is involved in the formation of cerebral edema. A recent retrospective study showed that the ALPS rate was lower in post-ischemic stroke patients and normalized over time, suggesting an impairment of glymphatic flow that gradually improved (158). In ischemic stroke, the removal of substances accumulated along the perivascular spaces is also damaged (159). The evidence for depolarization of AQP4 is also contradictory. Some studies have shown that AQP4 knockout leads to less swelling of the brain after a stroke (160). In a recent study, mice lacking AQP4 showed no swelling for 15 min (31). Administration of an aquaporin 4 inhibitor also resulted in less brain swelling and apoptosis 3 and 7 days after stroke (161). Post-stroke glymphatic influx may be reduced by 60% in such animals (6). However, there are studies that indicate that brain swelling was similar in both groups of mice, although mortality and motor regeneration were better in the group devoid of aquaporin (162). This finding was supported by some studies in non-stroke and knockout animal models, which found that the transport of substances was similar in both groups (17) and even delayed (163). However, the role of AQP4 seems to be confirmed by a recent study which showed that it can be used as a therapeutic target. Inhibition of calmodulin, which is responsible for the appropriate polarization of aquaporin, in a damaged core in rats led to inhibition of ablated edema and accelerated functional regeneration (164).

In preclinical studies, it was found that after the subarachnoid hemorrhage (SAH) there is a disturbance of glymphatic flow (156, 165) and loss of AQP4 polarization (165). Using GD-DTPA under MRI control to assess glymphatic flow in a rat model, it was noticed that AQP4 knockout promotes the development of edema after SAH, increases water accumulation in the brain and worsens neurological deficits (83). Disorder of glymphatic flow after SAH was also confirmed in a non-human primate model (166). The brain tissue factor (167) may be involved in this process, although the role of AQP4 (168) seems to be of key importance so far. Further research may bring us closer to the use of glymphatic system components as a therapeutic target, which has already been confirmed in a mouse model using pituitary adenylate cyclase-activating polypeptide (169), or nimodipine (170).

### 7.6. Central nervous system tumors and neoplasms

The role of the glymphatic system in the formation of edema after tumor development has also been postulated. A series of recent studies have measured glymphatic activity by MRI DTI-ALPS in patients with meningiomas, gliomas and tumor metastases. An inverse correlation was found between the ALPS index and the development of peritumoral brain edema (PTBE). It has been suggested that higher glymphatic function facilitates fluid removal and prevents swelling (158). A lower ALPS index was also observed in patients with wild-type IDH1 gliomas (compared to the IDH1 mutant) and gliomas with greater peritumoral edema. It may be related to the greater glymphatic impairment in this type of disease, or it may be due to the rapid growth time of wild-type glioblastoma (171). Lower ALPS was also observed in metastatic cancer patients who developed larger PTBE. An increase in ADC was also observed, possibly mediated by AQP4 (172). These studies confirm that the dysfunction of the glymphatic system can also be observed in CNS neoplasms.

### 7.7. Other medical conditions

Recently, glymphatic system dysfunction has been shown to be associated with juvenile myoclonic epilepsy. The mean ALPS index was lower in the group of sick patients. It also decreased with age (173). The same relationships were observed in patients with status epilepticus (174). A similar relationship–excluding the age of the patients–was observed among patients suffering from isolated REM sleep behavior disorders (175). The weakening of the function of the glymphatic system was also detected in multiple sclerosis. A retrospective study showed that lower perivascular space diffusion was associated with more severe clinical disability and longer disease duration. Patients with progressive disease showed lower diffusion compared to patients suffering from relapsing–remitting type (176). It has also been shown that extended PVS is associated with Huntington’s disease and that their burden affects the severity of the disease (177). Glymphatic system dysfunction has also been associated with epilepsy or simple febrile seizures (178).

## 8. Summary and discussion

This review presents the factors that influence the activity of the glymphatic system–the unique cleansing system of the brain. In recent years, a great deal of experimental evidence has emerged that it is an important element in keeping the brain healthy. It has been shown to be associated with neurodegenerative diseases and the aging process. Glymphatic activity depends on various factors. It is stimulated by the sleep state, especially in the 3rd and 4th phase of NREM, when delta waves are observed in the EEG. The lateral position during sleep also improves the flow of fluids through the glymphatic system. Clearly, sleep deprivation has the opposite effect. By taking care of the right amount and good quality of sleep, we contribute to proper glymphatic activity. Although the influence of the state of arousal on the activity of the brain’s lymphatic system is well documented, circadian fluctuations probably also play a role. There are many modifiable factors that affect the increase or decrease of its effectiveness. Omega-3 polyunsaturated fatty acids, intermittent fasting and physical activity improve glymphatic activity. Alcohol consumed in small amounts has a similar effect, while its intake in large amounts interferes with this transport. Stress and the coexistence of arterial hypertension also have an inhibitory role.

The first studies on the glymphatic system in humans have also been carried out. It has been shown that the assessment of glymphatic activity can contribute to a better diagnosis of neurobiological diseases and even to the improvement of therapeutic results. There are several imaging methods that can be used to assess glymphatic activity, such as MRI DTI-ALPS, rsfMRI BOLD, EPVS assessment, and lymphatic flow in the meninges. Further research is likely to contribute to the development of these research methods. It has already been proven that DTI-ALPS can be a biomarker of cognitive disorders (101–103) or predict the type of mutation in patients with glioblastoma (172). However, there are many hypotheses that should be tested in the future, such as the use of this indicator to assess the early stage of dementia or the differentiation of parkinsonism. Although it is a widely used method in studies of the glymphatic system, its accuracy should be confirmed and validated in future studies. Another element of the glymphatic system that raises high hopes is aquaporin 4. Restoring its polarity may be an important therapeutic point, which has been confirmed in preclinical studies. However, we still do not know everything about its role in the brain, such as its exact effect on cerebral edema following ischemic stroke. An interesting prospect also seems to be the use of the glymphatic system for better distribution and delivery of drugs to the CNS (68). The concept of the glymphatic system sheds new light on the pathophysiology of various neurological disorders, including Alzheimer’s disease. So far, no effective method of treating this disease has been found. Understanding the cleansing mechanism of the brain and the factors influencing it allows for the creation of lifestyle recommendations that will improve the functioning of the glymphatic system and will prevent or slow down the development of this disease, as well as other neurogenerative diseases. In the future, the brain’s lymphatic system may become a target for new generations of drugs and therapeutic interventions.

To make this possible it is necessary to develop research that will help learn more about the glymphatic system and respond to the current controversy (179). It is important to improve the strategy of the experiments conducted by researchers. It may be important to define standard measures and methods so that there is no contradiction between research groups, based on the adoption of a different methodology. The development of the imaging methods used (180) is a chance for a better understanding of the glymphatic system in the future. *In vivo* T1 mapping may be useful, e.g., for the quantification of glymphatic activity and drainage of deep and superficial lymph nodes (67). Neuroimaging can help understand the physiology of the brain’s lymphatic system in experiments with live animals and living people.

At the same time, more human research is needed. While rodents provide various data and are a good research model, and reports from human experiments appear to corroborate animal research data, more research projects that focus on humans are needed. Human brain function can be more complex and can be regulated in a number of ways. Different age groups need to be researched to understand brain behavior at different ages. An important perspective that has not been taken up so far is the focus on the glymphatic system in adolescents, who increasingly indicate sleep problems. This is especially true because the loss of sleep in adolescence causes cognitive decline. In their context, alcohol consumption, diet and exposure to stress are also important. This affects their health and promotes the development of dementia in the future. Gender research is also an interesting new perspective.

All the factors mentioned in this review provide an interesting perspective and have a chance to be used to modify glymphatic activity in the future. At the moment, however, they are insufficiently researched, so further experiments are needed. Studying non-modifiable factors can help spot people with lymphatic problems earlier, and learning about modifiable factors can help you change your life to a healthier one. Glymphatic activity, i.e., proper cleansing of the brain, largely depends on our daily choices. Furthermore clinical studies show that the glymphatic system is associated with many disease entities. This means that it plays a key role in the physiology of the whole brain, linking many pathophysiological pathways of individual diseases. Therefore, understanding its operation and influence on its activity can bring breakthroughs in neurology and neurosurgery. If subsequent studies confirm the results obtained in various disease entities, and the development of neuroimaging techniques continues, we cannot rule out that in a few years the “glymphogram” will become part of basic neurological care.

## Author contributions

AG: conceptualization, first draft preparation, and visualization. SS and DK: review and editing and supervision. All authors contributed to the article and approved the submitted version.

## Conflict of interest

The authors declare that the research was conducted in the absence of any commercial or financial relationships that could be construed as a potential conflict of interest.

## Publisher’s note

All claims expressed in this article are solely those of the authors and do not necessarily represent those of their affiliated organizations, or those of the publisher, the editors and the reviewers. Any product that may be evaluated in this article, or claim that may be made by its manufacturer, is not guaranteed or endorsed by the publisher.
